# Potential Agents against Plasma Leakage

**DOI:** 10.5402/2011/975048

**Published:** 2011-04-03

**Authors:** Jeanne Adiwinata Pawitan

**Affiliations:** Department of Histology, Faculty of Medicine, University of Indonesia, Jl. Salemba 6, Jakarta 10430, Indonesia

## Abstract

Shock due to severe plasma leakage may happen in infectious diseases such as severe dengue and sepsis due to various bacterial infections, which may be deleterious and may lead to death. Various substances and proteins are known to modulate the effects of proleakage mediators and counteract the deleterious effect of plasma leakage. Some of the various substances and proteins such as focal adhesion kinase (FAK), the Rho GTPases, protein kinase A, and caveolin-1 have dual actions; therefore they are not suitable for therapy. However, sphingosine 1phosphate and its receptor agonists, Angiopoetin-1, Slit, and Bbeta15–42 may be promising.

## 1. Introduction

Shock due to severe plasma leakage may happen in infectious diseases such as severe dengue [1], and sepsis due to various bacterial infections [2], which may be deleterious and may lead to death. Plasma leakage is caused by a substantial increase in endothelial permeability that is mainly due to loosening of interendothelial junction and focal adhesion due to host's response to infectious pathogens. Loosening of interendothelial junction is triggered by proleakage mediators such as thrombin, bradykinin, histamine, reactive oxygen species (ROS), vascular endothelial growth factor (VEGF), tumor necrosis factor alfa (TNF*α*), and bacterial lipopolysaccharides (LPS) that are alternatively called as endotoxin [3]. Currently, proteins that are involved in the interendothelial tightening or loosening events are accumulating. Therefore, this review will address interendothelial junction, focal adhesion, and various substances and proteins that may modulate the effects of proleakage mediators to counteract the deleterious effect of plasma leakage. Finally, future clinical application will be discussed.

## 2. Interendothelial Junction and Focal Adhesion

**Figure 1:**
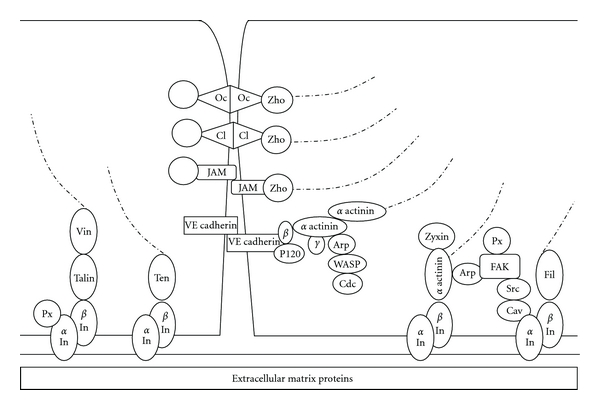
Interendothelial junctions and focal adhesions Oc = occludin, Cl = claudin, JAM = junctional adhesion molecules, Zho = zonula occludens proteins (zho-1), VE cadherin = vascular endothelial cadherin, *β* = *β* catenin, *γ* = *γ* catenin, p120 = p120 catenin, Arp = Arp2/3, WASP = Wiskott-Aldrich syndrome protein, Cdc = Cdc42, Vin = vinculin, Px = paxillin, *α*In = *α* integrin, *β*In = *β* integrin, FAK = focal adhesion kinase, Cav = caveolin-1, Fil = filamin, Ten = tensin, – · – · – · – = actin. Oc, Cl, JAM, and Zho are part of tight junctions, while VE cadherin, *α*, *β*, *γ*, p120 catenin are part of adhering junctions that can be modulated by Arp, WASP, and Cdc.

Inter-endothelial junctions (IEJs) responsible for the occurrence of plasma leakage are tight junctions (TJs) and adhering junctions (AJs) that form intercellular zipper-like structures between two cell borders. Tight junctions are composed of claudins, occludins, and junctional adhesion molecules (JAMs), that are connected to actin cytoskeleton by zonula occludens protein (ZO-1), while AJ are composed of calcium ion-dependent vascular endothelial- (VE-) cadherin that are linked to cytoskeletal actin through catenins, namely, *α*, *β*, *γ*, and p120-catenins [3] (Figure 1). 

In addition, endothelial cell membrane contains integrin that is a transmembrane protein, which forms focal adhesion to subendothelial extracellular matrix (ECM) proteins that consist of collagen IV, fibronectin, laminin, entactin, chondroitin sulfate, heparan sulfate, perlecan, syndecan, thrombospondin and secreted protein acidic and rich in cysteine (SPARC). Subendothelial ECM forms a highly elastic basement membrane with great tensile strength [3] (Figure 1). 

Further, integrin also directly or indirectly binds other focal adhesion associated proteins, that is, talin, vinculin, *α* actinin, paxillin, filamin, zyxin, and tensin [3, 4], and via these proteins are connected to cytoskeletal actin (Figure 1). These focal adhesion proteins interact with IEJ proteins. Therefore, TJs, AJs, and focal adhesions provide adhesive strength between adjoining cells that provide barrier functions [3].

When the IEJs are established, platelet endothelial cell adhesion molecule-1 (PECAM-1) rapidly occupies these junctions, and prevents the expression of leukocyte adhesion molecules such as E-selectin, and thus prevents leukocyte transmigration through the IEJs [5].

Proleakage mediators may interact with TJ, AJ, or ECM proteins and stimulate the pathway that cause loosening of IEJ, and ends up in plasma leakage. However, restoration of barrier function will usually follow due to substances and proteins that counteract the proleakage mediators and therefore are involved in the tightening and restoring of the IEJ [3].

## 3. Substances and Proteins to Counteract Proleakage Mediators

Various substances and proteins may counteract proleakage mediators and restore barrier functions; they are focal adhesion kinase (FAK), caveolin-1, Cdc42 and Rac, cAMP, protein kinase A (PKA) and adenylate cyclase, sphingosine 1phosphate (S1P) and its receptor agonist, angiopoetin-1 (Ang-1), Slit, and Bbeta15–42.

### 3.1. Focal Adhesion Kinase and Its Associated Proteins

Focal adhesion kinase is a protein with tyrosine kinase activity that regulates actin dynamics, binds to integrins, and is responsible for focal adhesion to ECM. In addition, FAK binds a triple enzyme called Trio that interacts with RhoA and Rac and is responsible for the inhibition of RhoA activity at focal adhesions, which protect against barrier disruption [3, 6, 7]. 

RhoA is one of the Rho-family small GTPases (Rho GTPases), which has dual action, that is, at basal expression level, it plays a role in intrinsic barrier protection at cell margins, but thrombin-induced increased activity of RhoA causes barrier disruption at focal adhesions [8].

**Figure 2:**
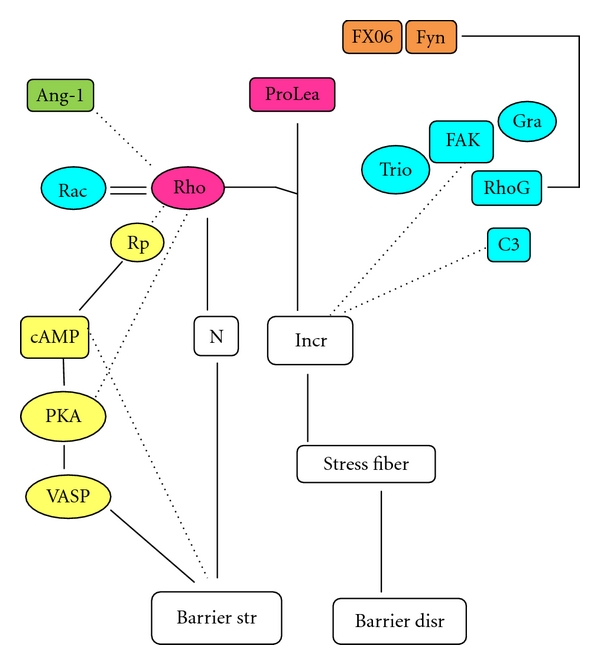
The role of focal adhesion kinase, its associated proteins, Rho GTPases, cAMP, angiopoetin-1, and Bbeta15–42 peptide in barrier maintenance. FX06 = Bbeta15–42 peptide, Ang-1 = angiopoetin-1, Prolea = proleakage mediators, Rho = Rho A, FAK = focal adhesion kinase, Gra = GTPase regulator associated with FAK (GRAF), RhoG = 190Rho GTPase activating protein (190RhoGAP), C3 = C3 toxin, N = normal level, Incr = increased level, Rp = Rap-1, PKA = protein kinase A, VASP = vasodilator stimulated phosphoprotein, Barrier str = barrier strengthening, Barrier disr = barrier disruption, solid line = contributing factor, and dashed line = inhibiting factor. Various substances are involved in barrier strengthening/disruption. Some substances have different effects that depend on the presence or the absence of proleakage mediators (normal condition); for instance, Rho contribute to barrier strengthening in normal condition, but contribute to stress fiber formation in the presence of proleakage mediators.

Thrombin-activated RhoA plays a role in actin stress fiber formation; and contraction of the stress fibers may disrupt the focal adhesion, and thus disrupt barrier function [8]. However, RhoA activation due to nonfunctioning FAK is inhibited by C3 toxin. Therefore, C3 toxin may restore endothelial barrier function due to nonfunctioning FAK [3]. Studies showed that FAK induced inhibition of RhoA activity needed cooperation of FAK-associated proteins, that is, GTPase regulator associated with FAK (GRAF) and 190Rho GTPase activating protein (190RhoGAP) [3, 9, 10] (Figure 2). 

Overall, FAK and its associated proteins (GRAF and 190RhoGAP) together with Trio and C3 toxin are supposed to restore increased vascular permeability due to proleakage mediators such as thrombin, histamine, and VEGF by recruiting focal adhesions [3, 11]. However, FAK is also required for thrombin-induced cell contraction that ends up in barrier disruption [12, 13].

### 3.2. Caveolin-1

Caveolin-1 is one of the caveolae membrane-associated regulatory proteins. Moreover, caveolin-1 has important function in signal transduction as well as molecular transport such as endocytosis and transcytosis across endothelium [14]. Integrin-caveolin-1 interaction is supposed to support endothelial barrier function [3]. Caveolin-1 binds integrin (Figure 1) [3, 15], and caveolin-1 expression inhibition causes an increase in endothelial paracellular permeability [16] due to suppression of integrin signaling and decrease in focal adhesion formation [3]. However, caveolin-1 regulates VEGF-induced VEGFR2 autophosphorylation that is followed by downstream signaling [14], which may trigger plasma leakage. Regulation of VEGF-induced VEGFR2 autophosphorylation is supposed to be due to compartmentalization of certain signaling molecules [14].

### 3.3. Cdc42 and Rac

Cdc42 and Rac are another Rho GTPases. Studies showed that in endothelial cells, Cdc42 and Rac regulated the formation of membrane ruffle associated actin filaments [17], which may induce the formation of IEJs, especially formation of AJ [3]. Moreover, Cdc42 and rac function is regulated by FAK [18] that causes actin polymerization [19], and AJ assembly [20]. Regulation by FAK involves other proteins, that is, G protein-coupled receptor kinase interactor-1 (GIT-1) and p21-activated kinase-interacting exchange factor (PIX) [3, 21].

 In addition, Cdc42 and Rac regulate actin-myosin interaction through p21-activated kinase (PAK) that inhibits myosin light chain kinase (MLCK) activity [22]. However, PAK has dual action, as constitutively active PAK can phosphorylate myosin light chain (MLC) and causes endothelial contraction, and thus barrier disruption [22, 23].

**Figure 3:**
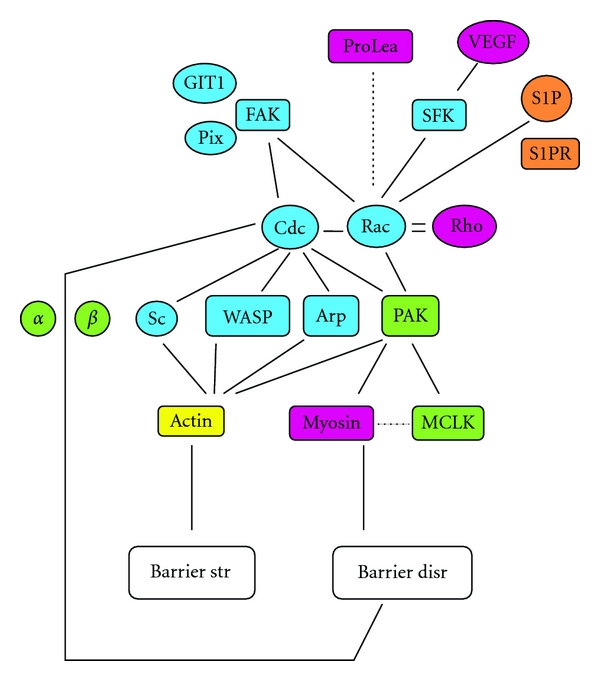
The role of Rho GTPases and sphingosine 1phosphate in barrier maintenance. Prolea = proleakage mediators, VEGF = VEGF-165 (the predominant isoform of VEGF A), GIT 1 = G protein-coupled receptor kinase interactor-1, Pix = p21-activated kinase-interacting exchange factor, FAK = focal adhesion kinase, SFK = Src family kinase, S1P = sphingosine 1phosphate, S1PR = sphingosine 1phosphate receptor, Cdc = Cdc42, Rho = RhoA, *α* = *α* catenin, *β* = *β* catenin, Sc = Scar, WASP = Wiskott-Aldrich syndrome protein, Arp = Arp2/3, PAK = p21-activated kinase, MCLK = myosin light chain kinase, Barrier str = barrier strengthening, Barrier disr = barrier disruption, solid line = contributing factor, and dashed line = inhibiting factor. The various Rho GTPases and S1P have dual role in barrier maintenance.

Another possible targets of Cdc42 are Wiskott-Aldrich syndrome protein (WASP), WASP family verprolin-homologous (WAVE) that is alternatively called Scar), and Arp2/3 that are activators of actin polymerization, which might facilitate AJ formation [24, 25] (Figure 3). 

Thrombin induced plasma leakage due to AJ disassembly is usually transient, and is followed by Cdc42 delayed activation that will restore the barrier function due to AJ reassembly [3, 20].

However, Cdc42 and rac have dual actions. Besides facilitating the formation of IEJs, cdc42 may increase vascular permeability by loosening AJ due to loosening *α*-*β* catenin interaction [3, 20] (Figure 3). Rac is required to maintain barrier integrity, as proleakage mediators such as thrombin decreases Rac activity and causes imbalance in the proportion of activated rac versus RhoA that ends up in plasma leakage. However, another proleakage mediator, namely, VEGF-165 (the predominant isoform of VEGF A) causes Src family kinase (SFK) dependent activation of Rac-1 that ends up in increased permeability [26, 27].

### 3.4. cAMP, PKA, and Adenylate Cyclase

In endothelial cells, cAMP is synthesized by adenylate cyclase and controlled by phosphodiesterase. On endothelial barrier, cAMP shows dual action, that is, barrier disruption and barrier protective action [3]. Increase in cytosolic cAMP level due to *Pseudomonas aeruginosa* EcoY disrupts the endothelial barrier [28], while in the presence of permeability increasing mediators, cAMP may strengthen endothelial barrier through 2 mechanisms, that is, the PKA dependent and PKA independent mechanisms [3].

In the PKA-dependent mechanism cAMP activates PKA that prevents RhoA activation [29], and thus inhibits endothelial contraction [3]. In addition, PKA phosphorylates vasodilator stimulated phosphoprotein (VASP), which causes occludin, JAM, and ZO-1 binding and promotes stabilization of IEJ [3]. In the PKA independent mechanism, cAMP increases Rap 1 (a member of the Ras family) [30] that induces phosphorylation of Rho A [31], followed by decreasing RhoA activity, thus strengthen the IEJs [3] (Figure 2).

### 3.5. S1P and S1P Receptor Agonist

Sphingosine 1phosphate is synthesized by sphingosine kinase and degraded by either sphingosine phosphatase or sphingosine lyase, and the three enzymes regulate S1P plasma level [32]. Platelets are the primary storage of S1P, as platelets lack the degrading enzymes. Endothelial cells have S1P receptors and might be continuously exposed to platelet released S1P that is bound to albumin, at a normal serum level of 250 to 500 nM [3]. 

The effect of normal level of S1P on endothelium is to maintain the IEJs. Further, in thrombin-induced barrier dysfunction, S1P accelerate the recovery of barrier function in vitro [33, 34]. However, at high concentration (5 *μ*M) S1P activates RhoA and stress fiber formation, thus disrupt the IEJs [35].

The mechanism for the maintenance of IEJs by physiological level of S1P requires binding to S1P receptors, which are G protein-coupled receptors and are members of the previously called endothelial differentiation gene (edg) family of receptors, namely, edg-1or S1P1 and edg-3 or S1P3 receptors [33, 36]. Further, it involves rac, as well as Rho kinase-dependent PAK activation that plays a role in cortical actin assembly due to phosphorylation of cofilin, which is an actin filament regulatory protein (Figure 3). In addition, tyrosine kinase activation is involved in cortical cytoskeletal rearrangement [33], followed by assembly of AJ [3, 33], and focal adhesion complex (FAC) [35–37], which strengthen the IEJs [3].

FTY720-P is a sphingosine kinase-mediated phosphorylation product of the potent immunosuppressive agent FTY720. FTY720-P is regarded as agonist of S1P receptor, as it has a high affinity for G protein-coupled S1P receptor. Upon binding to the receptor on endothelial cells, it mediates Akt phosphorylation followed by AJ assembly. In vitro, FTY720-P was shown to counteract VEGF-induced increased permeability, and in vivo, FTY720 administration strongly blocked VEGF-induced plasma leakage [36].

### 3.6. Angiopoetin-1

Angiopoetin-1 and VEGF are important regulators of embryonic and postnatal neovascularization. In later stages of embryonic angiogenesis Ang-1 cooperate with VEGF in inducing the formation of mature endothelial barrier [3]. Further, Ang-1 opposes proleakage-mediator-induced, such as VEGF-induced [38], as well as thrombin- [3], platelet activating factor (PAF)-, bradykinin- and histamine-induced [39] endothelial permeability increase. 

**Figure 4:**
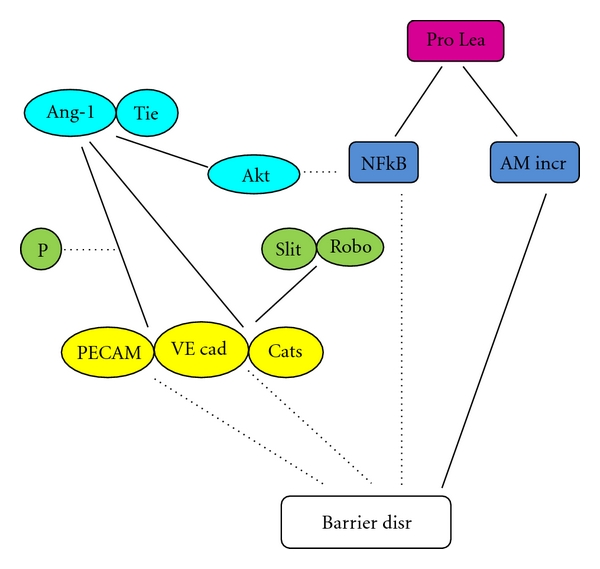
The role of angiopoetin-1 and slit in barrier maintenance Ang-1 = angiopoetin-1, Tie = Tie2 receptor, Prolea = proleakage mediators, P = phosphorylation, Akt = phosphatidylinositol 3′-kinase/Akt pathway, Robo = Roundabout receptor, NF*κ*B = nuclear factor kappa B, AM incr = adhesion molecule increase, PECAM = PECAM-1, VEcad = VE-cadherin, Cats = catenins, Barrier disr = barrier disruption, solid line = contributing factor, and dashed line = inhibiting factor.

The mechanisms of Ang-1-induced permeability inhibition are supposed via the inhibition of RhoA pathway (Figure 2) [3]. Ang-1maintain the localization of PECAM-1 into IEJs, and counteract proleakage-mediator-induced phosphorylation of PECAM-1 and VE-cadherin and also restore the impaired association between catenins and VE-cadherins, and therefore repair the impaired barrier integrity due to proleakage mediators [5] (Figure 4).

Moreover, Ang-1 suppresses VEGF-induced upregulation of intercellular adhesion molecule-1 (ICAM-1), vascular cell adhesion molecule-1 (VCAM-1), and E-selectin expression, followed by suppression of leukocyte adhesion to endothelial cells and transmigration through IEJs, and finally counteract VEGF induced plasma leakage by maintaining barrier integrity. Ang-1 suppression of those adhesion molecules is supposed to be mediated by binding to its receptor, namely, Tie2 receptor that strongly activates the phosphatidylinositol 3′-kinase/Akt pathway [40] (Figure 4). The pathway suppress nuclear factor kappa B (NF-*κ*B) that is activated upon VEGF binding to its receptor, namely, the VEGF receptor2 (VEGFR2), that is alternatively called as fetal liver kinase-1(Flk-1)/kinase insert domain containing receptor (KDR) [40].

### 3.7. Slit

Slit is the soluble ligand of the Roundabout (Robo) receptor that was initially found in axons, but later, an endothelial specific Robo that is called Robo4 was found. In axons, Slit-Robo is part of a signaling pathway that is involved in axon guidance, while in endothelial cells, Slit-Robo maintains vascular barrier in the mature vascular network, by inhibiting endothelial hyperpermeability that is induced by proleakage mediators [41].

The mechanism of barrier maintenance involves interaction of Slit-Robo4 with a family of intracellular adaptor proteins, namely, paxillin and Hic-5. This interaction recruits GIT1 that is one of the Arf-GAPs and causes inactivation of Arf (a small GTPase) followed by inhibition of Rac activation, which leads to inhibition of plasma leakage [41].

In VEGF-165-induced plasma leakage, slit2-Robo4 inhibit Rac-1 activation due to inhibition of SFK-Rac signaling, namely, phosphorylation of SFKs, including Fyn, Yes, and Src, which occurred downstream of the VEGFR2 that is autophosphorylated upon binding to VEGF-165 [42]. In addition, Slit-Robo4 inhibits VEGF-165 induced plasma leakage by blocking the activation of Arf6 [41].

The IEJ components that are involved in slit-Robo4 inhibition of plasma leakage are VE-caherin and p120 catenin at AJs. Slit-Robo4 prevents dissociation of VE-cadherin from p120 catenin, which happens in plasma leakage, and therefore prevents AJ disruption (Figure 4) by preventing displacement of VE-cadherin to the interior of the cell [2, 43].

### 3.8. Bbeta15–42

Bbeta15–42 (alternatively called as FX06) is a fibrin-derived natural peptide that occurred after fibrin degradation by plasmin. The peptide is recently regarded as a signaling molecule that significantly reduces plasma leakage in animal models, as it stabilizes endothelial barriers [13].

The mechanism involves activation of Rac and prevention of RhoA activation. Further, the peptide restores the imbalance in activated Rac-RhoA and counteracts thrombin-induced stress fiber formation and myosin light chain phosphorylation, thus preventing cell contraction and the loosening of IEJs. Prevention of RhoA activation is mediated through Fyn that dissociates from VE-cadherin at AJs, upon exposure to the peptide. Subsequently, Fyn associates with p190RhoGAP and prevents RhoA activation (Figure 2). Moreover, prevention of cell contraction also involves FAK, as the peptide causes diffuse distribution of FAK in the cytosol, instead of localization of FAK at the tip of stress fibers that happens in the presence of thrombin alone [13].

## 4. Future Clinical Application

As some of the various substances and proteins such as FAK, the three Rho GTPases (RhoA, Cdc42, and Rac), cAMP, PKA, and caveolin-1 may have dual actions, they are not suitable for the therapy of plasma leakage. On the other hand, S1P and its receptor agonists, Ang-1, Slit, and Bbeta15–42 may be promising to be studied further. 

Various diseases may involved plasma leakage in various degrees and may benefit from the substances that can prevent the occurrence or control the occurring plasma leakage. Plasma leakage may happen in various conditions, such as respiratory distress syndrome due to sepsis from various etiologies, shock that is followed by multiorgan failure due to cytokine storm as that happens in various acute infections, conditions with tissue edema, and chronic peripheral vascular disorders associated with diabetes [36]. Therefore, those substances need to be evaluated, first in animal models for those various conditions, followed by clinical trials to confirm their use in human, if studies in animal models show promising results.

### 4.1. S1P and Its Receptor Agonists

An animal study in mice showed that S1P lacking mutant mice benefited from pretreatment with S1P1 receptor agonist (AUY954) or wild-type erythrocytes, which restored plasma S1P levels, before induction of plasma leakage. However, wild-type mice did not benefit pretreatment with the S1P1 receptor agonist [44]. Another study that used extravasation of FITC-dextran as a measure of plasma leakage showed that administration of S1P receptor agonist (FTY720) orally before inducing plasma leakage by VEGF, potently reduced plasma leakage compared to controls [36].

However, in the animal studies S1P and its receptor agonist were administered before induction of plasma leakage, thus preventing the occurrence of plasma leakage. Whether they can control occurring plasma leakage by restoring the endothelial barrier remains to be established.

### 4.2. Ang-1

An animal study in mice showed that Ang-1 delivery through adenoviral transduction of Ang-1 gene followed by proleakage mediator challenge caused plasma leakage reduction from the venules due to decrease in the number and size of endothelial gaps [45]. Further, in a study on patients with septic shock, Ang-1 level was significantly lower compared to patients with sepsis without shock [46], and in another study, Ang-1 level in patients with sepsis was lower compared to healthy controls [47]. These results suggest that Ang-1 administration may be beneficial to control plasma leakage in sepsis, especially septic shock. 

However, whether Ang-1 really can be used to treat plasma leakage and not only prevents it from occurring as was shown in the animal study remains to be established.

### 4.3. Slit

A study on 3 kinds of animal models, that is, for gram-negative bacterial pneumonia, intraabdominal sepsis, and H5N1 influenza virus infection showed that intravenous Slit administration before the induction of disease strongly reduced plasma leakage in the lung and other organs, and decreased mortality in the animals, without any effect on the pathogen-induced cytokine storm [43]. As in the case of S1P and Ang-1, Slit was administered before induction of the various diseases, therefore, studies on already infected animals need to be conducted to reveal whether Slit only prevents, or is able to treat plasma leakage. The implication if it only prevents, it should be administered early in individuals at risk for severe disease, before plasma leakage is prominent.

### 4.4. Bbeta15–42 (FX06)

In an animal model for LPS pneumonitis with plasma leakage, FX06 treatment directly and 60 minutes after LPS intranasal instillation significantly reduced plasma leakage and inflammation. In another animal model for dengue shock syndrome, day 3 after infection administration of FX06 significantly reduced plasma leakage through capillaries in the lungs and intestines, reduces hemoconcentration and fibrinogen consumption, and improved survival, but did not affect viral loads in serum, liver, and brain [13].

FX06 has entered phase I clinical trial that involved 30 male healthy volunteers in a randomized double blind placebo controlled, parallel group study to test the pharmacokinetics and safety of single ascending doses, and was proven safe [48]. Further, FX06 was used in a phase II clinical trial for patients with acute myocardial infarction with good results [49]. Whether FX06 has clinical benefit in plasma leakage due to various pathogens remains to be established.

## 5. Conclusion

S1P and its receptor agonists, Ang-1, Slit, and Bbeta15–42 showed promising results in preventing plasma leakage in animal studies. Their potentials to prevent and control occurring plasma leakage due to various diseases in human remain to be established.
